# MicroRNA Regulation of Bovine Monocyte Inflammatory and Metabolic Networks in an *In Vivo* Infection Model

**DOI:** 10.1534/g3.113.009936

**Published:** 2014-01-23

**Authors:** Nathan Lawless, Timothy A. Reinhardt, Kenneth Bryan, Mike Baker, Bruce Pesch, Duane Zimmerman, Kurt Zuelke, Tad Sonstegard, Cliona O’Farrelly, John D. Lippolis, David J. Lynn

**Affiliations:** * Animal and Bioscience Research Department, Animal and Grassland Research and Innovation Centre, Teagasc, Grange, Dunsany, County Meath, Ireland; † School of Biochemistry and Immunology, Trinity College, Dublin 2, Ireland; ‡ United States Department of Agriculture, Agricultural Research Services, National Animal Disease Center, Ames, Iowa 50010; § Iowa State University, DNA Facility, Molecular Biology Building, Ames, Iowa 50010; ** Australian Animal Health Laboratory, Commonwealth Scientific and Industrial Research Organisation, East Geelong Victoria 3219, Australia; †† United States Department of Agriculture, Agricultural Research Services, Beltsville, Maryland 20705-1350

**Keywords:** infection, innate immunity, RNAseq, microRNA, transcriptional networks, complex genetics, tolerance, complex immunity, resistance

## Abstract

Bovine mastitis is an inflammation-driven disease of the bovine mammary gland that costs the global dairy industry several billion dollars per year. Because disease susceptibility is a multifactorial complex phenotype, an integrative biology approach is required to dissect the molecular networks involved. Here, we report such an approach using next-generation sequencing combined with advanced network and pathway biology methods to simultaneously profile mRNA and miRNA expression at multiple time points (0, 12, 24, 36 and 48 hr) in milk and blood FACS-isolated CD14^+^ monocytes from animals infected *in vivo* with *Streptococcus uberis*. More than 3700 differentially expressed (DE) genes were identified in milk-isolated monocytes (MIMs), a key immune cell recruited to the site of infection during mastitis. Upregulated genes were significantly enriched for inflammatory pathways, whereas downregulated genes were enriched for nonglycolytic metabolic pathways. Monocyte transcriptional changes in the blood, however, were more subtle but highlighted the impact of this infection systemically. Genes upregulated in blood-isolated monocytes (BIMs) showed a significant association with interferon and chemokine signaling. Furthermore, 26 miRNAs were DE in MIMs and three were DE in BIMs. Pathway analysis revealed that predicted targets of downregulated miRNAs were highly enriched for roles in innate immunity (FDR < 3.4E−8), particularly TLR signaling, whereas upregulated miRNAs preferentially targeted genes involved in metabolism. We conclude that during *S. uberis* infection miRNAs are key amplifiers of monocyte inflammatory response networks and repressors of several metabolic pathways.

Bovine mastitis is an inflammation-driven disease of the bovine mammary gland that is associated with significant costs to the global dairy industry. In Europe this cost is estimated to be approximately €2 billion euro per year (Wells *et al.* 1998), with similar figures available in the United States (Jones and Bailey 2009). Causative agents of mastitis infection include, but are not limited to, coliforms (*Escherichia coli)*, Streptococci (*Streptococcus uberis*) and Staphylococci *(Staphylococcus aureus*). *S. uberis* is now ranked among the most prevalent mastitis-causing pathogens throughout Europe and North America (Reinoso *et al.* 2011; Ward *et al.* 2009).

Mastitis develops as bacteria entering the udder via the teat canal stimulate a pathological form of inflammation. Bacteria encounter epithelial cells lining the mammary gland stimulating a local inflammatory response that facilitates their transport across the epithelial barrier, where they are detected by resident immune cells, such as monocytes. Both cell types constitutively express surface pathogen recognition molecules such as Toll-like receptors (TLRs), enabling them to function in a sentinel capacity. Invasive *S. uberis* triggers TLR2 and TLR4 mobilizing local and systemic inflammatory mediators (Bannerman *et al.* 2004; Moyes *et al.* 2009). Typically, chemokines, interleukins (ILs), and tumor necrosis factor (TNF)-α initiate local physiological changes in vascular permeability, cell differentiation, and apoptosis. Concurrently, systemic innate immune changes provoke acute phase protein (APP) production, which is distributed systemically to suppress the spread of bacteria locally (Mitterhuemer *et al.* 2010). During this phase, immune cells are recruited to the point of infection (Rinaldi *et al.* 2010). Monocytes are released from the bone marrow into the circulatory system and eventually reach the mammary gland via chemokine ligand–mediated cell migration. There they differentiate into macrophage and dendritic cell populations (Dong *et al.* 2013; Shi and Pamer 2011). Neutrophils comprise the majority of immune cells in an infected gland during an infection. Neutrophils are tasked with directly clearing invasive bacteria via phagocytosis or neutrophil extracellular traps (NETS) and subsequently aid in resolution of inflammation (Lippolis *et al.* 2006; Reinhardt *et al.* 2013). Once recruited to the site of infection, monocytes and neutrophils orchestrate antimicrobial activity to control bacterial spread and resolve the infection (Dong *et al.* 2013; Serbina *et al.* 2008). Immune cells and other somatic cells can be detected in the milk of infected animals, and the counts of the number of such somatic cells per ml, called the somatic cell count, is an indicator of mastitis (Jones and Bailey 2009).

The local immune response in mammary tissues has been examined by several approaches both *in vivo* and *in vitro*. Candidate gene-based approaches and microarray technology have determined that more than 2000 genes spanning immunity, metabolism, and tissue remodeling are active during mastitis (Mitterhuemer *et al.* 2010; Moyes *et al.* 2009; Swanson *et al.* 2009). However, modest data are available examining transcriptional activity in either milk or blood monocytes from infected animals (Prgomet *et al.* 2005), and little is known regarding the role microRNAs (miRNAs) play in regulating these responses.

The miRNAs are small, noncoding RNAs that play a key role in the regulation of innate and adaptive immunity as posttranscriptional regulators of gene expression (O’Connell *et al.* 2010). They have been shown to regulate immune function in several cell types. Neutrophil senescence, for example, is regulated by a discrete miRNA repertoire (Ward *et al.* 2011). Naïve mouse B cells are indirectly regulated by miR-155 via histone deacetylase 4 repression, whereas naive CD4^+^ T-cell differentiation and function are regulated by global changes in miRNAs (Bronevetsky *et al.* 2013; Sandhu *et al.* 2012). In a recent study, we concluded that miRNAs likely play a key role in regulating the innate immune response in mammary epithelial cells to a bovine mastitis pathogen *in vitro* (Lawless *et al.* 2013).

Although miRNA expression is abundant in numerous bovine tissues, genome-wide studies elucidating the regulatory roles of miRNAs in bovine immunity are limited (Coutinho *et al.* 2007; Jin *et al.* 2009; Xu *et al.* 2009). Furthermore, no bovine studies to date have applied next-generation sequencing (NGS) to examine global miRNA expression in immunity and infection *in vivo*. In this study, we report a NGS approach to profile the expression of bovine miRNAs and mRNAs at multiple time points in milk and blood CD14^+^ monocyte cells isolated from Holstein Friesians infected *in vivo* with *S. uberis*, a causative agent of bovine mastitis.

## Materials and Methods

### Animals

Ten female Holstein Friesians in the middle of their first lactation period, aged between 26 and 30 months and between 3 and 5 months postpartum, were selected for this study. The trial was conducted at the USDA National Animal Disease Center (NADC) in Ames, Iowa. All animals had a medical history that was free from mastitis. The National Animal Disease Center’s Animal Care and Use Committee approved all procedures used in this study.

### Infection protocol

Five animals were infected via the teat canal of the right front quarter with approximately 500 colony-forming units (CFU) of a mastitis-causing pathogen, *S. uberis* 0140, in 10 ml saline. Five control animals were inoculated with saline only. Milk and blood samples were obtained from each animal at 0, 12, 24, 36, and 48 hr after infection (or mock infection) as described below. At each time point, rectal temperature, total volume of milk, somatic cell count, bacterial counts, ambient temperature, humidity, and additional observations were recorded for each animal. Bacterial counts were determined from 5-ml milk samples collected aseptically from the infected quarter. Milk was serially diluted in sterile phosphate-buffered saline and spread on blood agar plates and then incubated for 24 hr at 37°. After incubation, plates were examined for bacterial growth and CFUs per ml were determined.

### Cell extraction from milk

Milk was collected using a sterilized quarter milker from infected and control animals at each time point. The total volume of milk was noted; 5 ml milk was isolated for milk bacteriology. The remaining milk was then diluted into Hanks' Balanced Salt Solution (HBSS) without Phenol red, Mg, and Ca... plus 10 mM EDTA. The mixture was inverted several times, transferred to a 1-liter centrifuge bottle, and centrifuged in a fixed angle rotor at 10,000 × *g* for 30 min. After centrifugation, the supernatant was poured off and the pellets were resuspended in 150 ml HBSS plus 5 mM EDTA. The resuspended pellets were then transferred to fresh centrifuge tubes and spun at 2500 × *g* for 30 min. After the second spin, the supernatant was poured off and the pellet from each sample was resuspended in 20 ml RPMI 1640 plus 1 mM sodium pyruvate plus 2 mM L-glutamine plus 50 ug/ml gentamycin plus 10% FBS (cRPMI) (Sigma-Aldrich, Steinheim, Germany). The 20-ml cell suspension was divided into 2 × 10 ml aliquots in 15 ml conical centrifuge tubes. The cells were pelleted by centrifuge at 650 × *g*. After this spin, the pellets were pooled into 4 ml cRPMI and were labeled for cell sorting. The cells were counted by trypsin blue exclusion (Careforde, Chicago, IL) to determine the cell count.

### Cell extraction from blood

At each time point, animals were led into a crush, and 2×60 ml syringes of blood were extracted by venipuncture and immediately placed on ice. The total volume of blood and total number of cells in blood were determined using a hemocytometer. Blood was spun for 20 min at 1200 × *g*. The buffy coat was observed between serum and red blood cell phase and was removed. Contaminating red blood cells were lysed by adding 1 volume lysis solution (10.6 mM Na_2_HPO_4_ and 2.7 mM NaH_2_PO_4_) and inverting the tubes several times, immediately followed by adding half volume restore solution (10.6 mM Na_2_HPO_4_, 2.7 mM NaH_2_PO_4_, and 460 mM NaCl) and inverting the tubes. The buffy coat was then spun for 10 min at 650 × *g*, and the red supernatant was poured off. Cells were resuspended in red blood cell lysis solution and then restore solution again as described above. Cell were spun for another 5 min at 650 × *g* and resuspended in 5–10 ml media.

### Isolation of CD14^+^ monocytes by flow cytometry

Milk-derived and blood-derived CD14^+^ monocytes were isolated by fluorescence-activated cell sorting (FACS). Briefly, cells were labeled with monoclonal anti-bovine CD14 (Clone CAM36A; VMRD, Pullman, WA) and a PE-conjugated anti-mouse IgG1 antibody (Southern Biotechnology, Birmingham, AL). Labeled cells were separated based on fluorescence intensity using the BD FACS Aria Cell Sorting System (BD Biosciences, San Jose, CA). Cells with more than 95% purity were isolated from the milk and peripheral blood of each animal.

### mRNA extraction

The mirVana RNA Isolation Kit (Ambion, Austin, TX) was used to extract total RNA from FACS-isolated cell populations. Procedures were performed according to the manufacturer’s protocol (Supporting Information, File S1). RNA was quantified and integrity was confirmed using an Agilent RNA Kit on a 2100 Bioanalyzer platform (Agilent Technologies, Loveland, CO) (File S1).

### miRNA extraction

MicroRNA was extracted using mirPremier microRNA Isolation Kits (Sigma-Aldrich, Steinheim, Germany). Procedures were performed according to the manufacturer’s protocol (File S1). Small RNA was quantified using an Agilent small RNA Kit on a 2100 Bioanalyzer platform (File S1).

### mRNA library generation

One hundred indexed mRNA libraries (50 blood monocyte libraries and 50 milk monocyte libraries) were prepared for cluster generation using TruSeq v2 RNA sample preparation kits (Illumina, San Diego, CA). Procedures were performed according to the manufacturer’s protocol (File S1). The finished libraries were validated on an Agilent Bioanalyzer 2100 using an Agilent DNA-1000 chip (Agilent, Loveland, CO), at which point they were loaded for cluster generation. The samples were sequenced on an Illumina HiSequation 2000 at the Iowa State Sequencing Center (50-bp single-end). Infected and control samples (n = 100) were randomized across four flow cells (*i.e.*, three or four samples multiplexed per lane) to avoid confounding flow cell/lane effects (Auer and Doerge 2010). The barcode compatibility chart provided with the TruSeq RNA sample preparation kit was adhered to when pooling libraries. Fastq files were produced using the CASAVA 1.8 pipeline.

### mRNAseq analysis

One hundred fastq files were generated containing the sequencing data for each of the 100 mRNAseq libraries. The quality and number of the reads for each sample were then assessed using FASTQC v0.10.0 (http://www.bioinformatics.bbsrc.ac.uk/projects/fastqc/). Each sample was then put through a number of quality-control filters. First, reads were filtered using the fastq Illumina filter v0.1 (http://cancan.cshl.edu/labmembers/gordon/fastq_illumina_filter/), which removes reads from the fastq files that were flagged as not passing the Illumina CASAVA pipeline filters. Cutadapt v1.2 (http://code.google.com/p/cutadapt/) was used to trim the adaptors from reads when necessary. The remaining reads were then further filtered using the fastq quality filter package (http://hannonlab.cshl.edu/fastx_toolkit/) v0.0.13.2. When at least 70% of the bases had a Phred score <20, reads were removed. Reads passing all the aforementioned filters were also trimmed at their ends to remove low-quality bases (Phred score <20). Reads that were <20 nt after trimming were discarded. Reads that passed all quality-control steps were then aligned to the bovine genome (UMD3.1 assembly) (Zimin *et al.* 2009) using TopHat v2.0.8 (Trapnell *et al.* 2009), allowing one mismatch. Reads that did not uniquely align to the genome were discarded. HTSeq-count version 0.5.3p3 (http://wwwhuber.embl.de/users/anders/HTSeq/doc/overview.html) using the union model was used to assign uniquely aligned reads to Ensembl (v69) annotated bovine genes.

### Differential gene expression analysis

Data were normalized across libraries using the trimmed mean of M-values (TMM) normalization method (Bullard *et al.* 2010). The R (version 2.15.2) Bioconductor package EdgeR (v2.4.6) (Robinson *et al.* 2010), which uses a negative binomial distribution model to account for both biological and technical variability, was applied to identify statistically significant differentially expressed (DE) genes. Any samples that had less than five million uniquely aligning reads were removed from further analysis. Only genes that had at least one count per million in at least three samples were analyzed for evidence of differential gene expression. The analysis was undertaken using moderated tagwise dispersions. DE genes were defined as having a fold change in gene expression >1.5 and a Benjamini and Hochberg corrected FDR of <0.05 (Benjamini and Hochberg 1995).

### Hierarchical clustering

Hierarchical clustering of milk and blood mRNA normalized read counts were performed in the R (version 2.15.2) *hclust* package. Heatmaps were generated using the R *heatmap* package.

### Gene ontology and pathway analysis

The R (version 2.15.2) Bioconductor package GOseq (version 1.10.0), which corrects for gene length bias (Young *et al.* 2010), was used to identify overrepresented pathways using pathway annotation imported from the Kyoto Encyclopedia of Genes and Genomes (KEGG) (Kanehisa *et al.* 2007) database. KEGG disease pathways were excluded to focus the analysis on primary signaling pathways. Pathways were considered significantly overrepresented with an FDR <0.05. Pathway analysis was undertaken using Ensembl predicted human 1:1 orthologs of the bovine DE genes.

Additionally, we manually generated two pathway annotations that were of interest but not annotated in detail in KEGG: the “inflammasome” and “interferon” pathways. Gene IDs for these pathways were sourced from SA Biosciences (Qiagen) RT^2^ Profiler PCR Array Human Interferon and Receptors (PAHS-064A) and RT^2^ Profiler PCR Array Human Inflammasome (PAHS-097A) annotations. The interferon pathway consisted of 84 genes (Table S1) with expression controlled by or involved in cell signaling mediated by interferon ligands and receptors, whereas the inflammasome pathway consisted of 95 key genes (Table S1) involved in the function of inflammasomes, protein complexes involved in innate immunity, and general NOD-like receptor (NLR) signaling.

### Network analysis methods

To generate molecular interaction networks, the human 1:1 orthologs of bovine genes that were DE during at least one of the four time points in milk-isolated monocytes (MIMs) from *S. uberis*–infected animals were uploaded to InnateDB (www.innatedb.com) (Lynn *et al.* 2008). InnateDB is one of the most comprehensive databases of all human and mouse experimentally supported molecular interactions (>300,000 interactions in July 2013) but also specifically includes annotation on more than 19,000 manually curated human and mouse innate immunity relevant interactions, many of which are not present in any other database (Lynn *et al.* 2010). Networks were visualized using Cytoscape 2.8.2 (Shannon *et al.* 2003).

The network was analyzed using the cytoHubba plugin (Lin *et al.* 2008) for Cytoscape 2.8.2 (Shannon *et al.* 2003) to identify network hubs and bottlenecks using default parameters. The jActiveModules plugin (Ideker *et al.* 2002) in Cytoscape 2.8.2 (Shannon *et al.* 2003) was also used to identify high-scoring DE subnetworks (overlap threshold = 0.3; search depth = 3; number of modules = 5; “regional scoring” and “adjust score for size” were both enabled). The InnateDB pathway analysis tool was used to identify overrepresented pathways among module genes.

### miRNA library generation

One hundred indexed miRNA libraries (50 blood and 50 milk monocyte libraries) were also prepared for cluster generation and sequencing using the TruSeq Small RNA sample preparation kit. These miRNA libraries were prepared from the same samples that the mRNAseq libraries were prepared. Procedures were performed according to the manufacturer’s protocol (File S1). The finished libraries were validated on an Agilent Bioanalyzer 2100 using an Agilent DNA high-sensitivity chip (Agilent), at which point they were loaded for cluster generation. The samples were sequenced on an Illumina HiSequation 2000 (50-bp single-end). Infected and control samples (n =100) were randomized across three flow cells (*i.e.*, seven or eight samples multiplexed per lane) to avoid confounding flow cell/lane effects (Auer and Doerge 2010). Fastq files were produced using the CASAVA 1.8 pipeline. In a few cases, the sequenced miRNA libraries were found to be adaptor-contaminated. These libraries were repurified and resequenced. The list of resequenced samples can be found in Table S2.

### miRNAseq Analysis

Preliminary quality-control analysis of the 100 miRNAseq fastq files was again performed with FASTQC software v0.10.0 (http://www.bioinformatics.babraham.ac.uk/projects/fastqc/). Cutadapt v1.2 (code.google.com/p/cutadapt/) was then used to trim 3′ adaptor sequences. Reads that were shorter than 18 nucleotides after trimming were discarded. Trimmed reads were then further filtered using the fastq quality filter (http://hannonlab.cshl.edu/fastx_toolkit/) v0.0.13.2. Reads with at least 70% of the bases with a Phred score <20 were removed (Cock *et al.* 2010). Finally, reads passing all these filters were also trimmed at their ends to remove low-quality bases (Phred score <20). Reads that successfully passed filtering were aligned to the bovine genome (UMD3.1) using novoalign v2.08.03 in miRNA mode (http://www.novocraft.com) allowing one mismatch. Nonuniquely aligning reads were discarded. HTSeq version 0.5.3p3 (http://www-huber.embl.de/users/anders/HTSeq/doc/overview.html) using the union model was used to assign uniquely aligned reads to miRBase v19 miRNA annotation (Kozomara and Griffiths-Jones 2011). The sequencing data from this publication have been submitted to the NCBI GEO database and assigned the identifier (GSE51858).

### Differential miRNA expression analysis

Before assessing differential expression, miRNAseq count data were first normalized across libraries using either the trimmed mean of M-values (TMM) normalization method (Robinson *et al.* 2010) or the upper-quantile normalization (Bullard *et al.* 2010). Differential expression analysis of miRNAseq data has been shown to be sensitive to the normalization approach implemented (Garmire and Subramaniam 2012). To address this issue, we identified DE miRNAs in two alternatively normalized datasets: TMM-normalized (Robinson *et al.* 2010), upper-quantile normalized, and with no normalization. Only miRNAs that were identified as DE across all three datasets were considered further, *i.e.*, the differential expression of these miRNAs was robust to the normalization procedure (Lawless *et al.* 2013). Any samples that had less than two million uniquely aligning reads were removed from further analysis. The R (version 2.15.2) Bioconductor package EdgeR (v2.4.6) (Robinson *et al.* 2010) was applied to identify statistically significant DE miRNAs. The analysis was undertaken using moderated tagwise dispersions. DE miRNAs were defined as having a Benjamini and Hochberg corrected P value of < 0.05 (Benjamini and Hochberg 1995).

### Coexpression and target analysis of miRNA and mRNA data

To identify mRNAs that were potentially regulated by DE miRNAs in MIMs, we first sought to calculate Pearson correlations using the Apache commons Java statistics library between all DE miRNA expression in reads per million (rpm) and all mRNA expression (TMM normalized read counts) over the time course. A correlation matrix was constructed consisting of 26 (DE miRNAs) × 24,616 (all mRNAs) correlation coefficients. The resulting correlation matrix was then filtered to remove nonsignificant correlations (critical value for Pearson correlation for this matrix is r = −0.3116) and those inverse correlations that were not supported by miRanda-predicted miRNA-target pairs (miRanda v3.3a). The mRNAs predicted to be targeted (*i.e.*, had a significant anticorrelation relationship in the expression of the mRNA and the miRNA, plus a predicted seed target) by either upregulated or downregulated miRNAs were then selected for pathway analysis. Two-dimensional cluster analysis and visualization, using R version 2.15.3 *hclust* and *heatmap.plus* packages, were then applied to the filtered correlation matrix.

### Pathway analysis of predicted miRNA target genes

Target genes of DE miRNAs were submitted to InnateDB (Lynn *et al.* 2008) for pathway analysis. Genes were submitted in two groups, those that were targets of upregulated miRNAs and those that were targets of downregulated miRNAs. Significant pathways were calculated based on hypergeometric analysis; pathways of interest were defined as having a Benjamini and Hochberg corrected P value of < 0.05 (Benjamini and Hochberg 1995).

### Novel miRNA discovery

Using the software package miRDeep2 v0.0.5 (Mackowiak 2011), we examined whether milk/blood monocytes encoded for miRNAs not yet annotated in the bovine genome. We further parsed these data using a number of different parameters to identify those novel miRNAs that have the highest likelihood of being true positives as described previously (Lawless *et al.* 2013). Specifically, we identified those predictions when the mature and star stands were expressed with a minimum of five reads each; when miRDeep2 predicted that the miRNA had >90% probability of being a true positive; when the hairpin structure had a significant Randfold P value; and when the novel miRNA was independently predicted in two or more different miRNAseq samples.

## Results

### Kinetics of *S. uberis* infection *in vivo*

**Figure 1:**
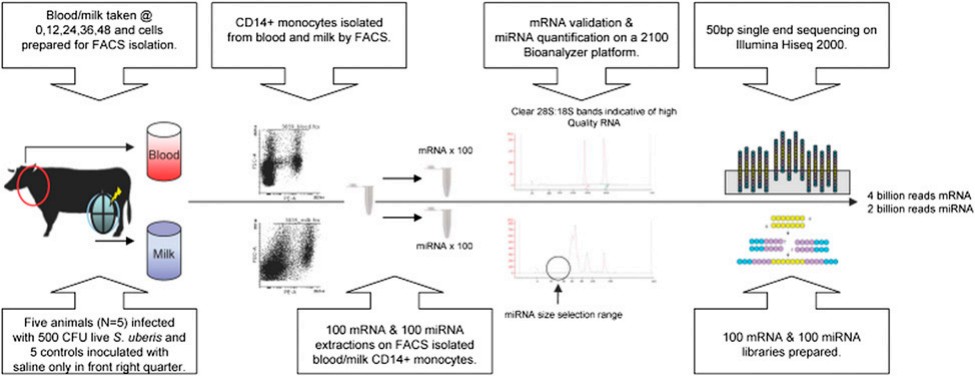
Overview of the experimental design. Five Holstein Friesian animals were infected via the teat canal of the right front quarter with approximately 500 CFU of a mastitis-causing pathogen, *Streptococcus uberis* 0140, in 10 ml saline. Five control animals were inoculated with saline only. Milk and blood samples were obtained from each animal at 0, 12, 24, 36, and 48 hr after infection (or mock infection). Milk-derived and blood-derived CD14^+^ monocytes were isolated by fluorescence-activated cell sorting (FACS) from each sample. mRNA and miRNA were extracted and 200 Illumina-compatible libraries were prepared for sequencing on a Hiseq 2000 machine. More than four billion mRNA reads and more than two billion miRNA reads were sequenced in total.

**Figure 2:**
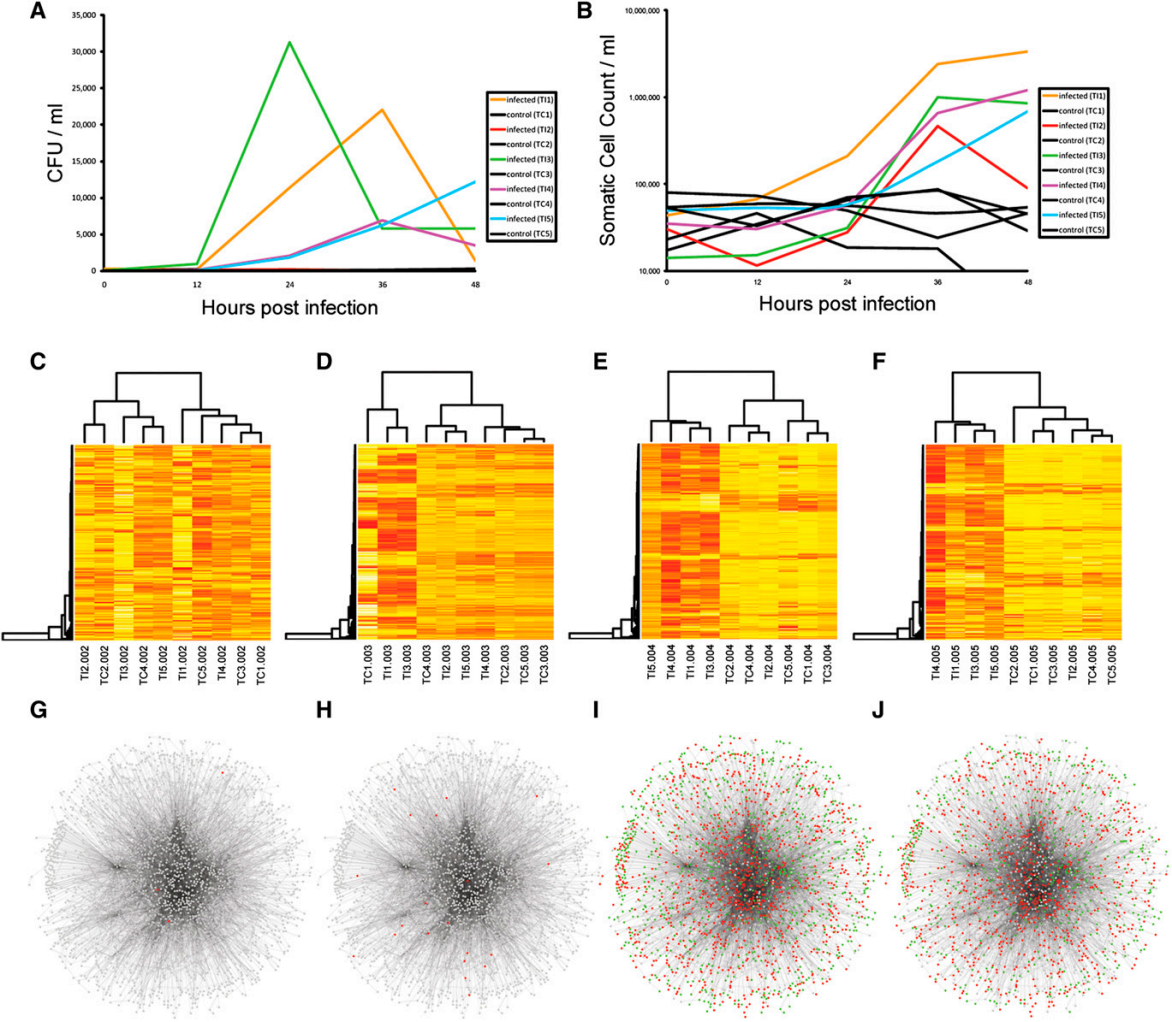
The response to infection. (A) The infection was monitored using recorded milk bacterial counts (CFU/ml) and (B) somatic cell counts (per ml) at each of the five time points (0, 12, 24, 36, and 48 hpi) for each animal (control and infected). Significant heterogeneity was observed in the CFU data among the infected animals. One infected animal (TI2) was observed to have only a very modest increase in bacterial counts. (C–F) Hierarchical clustering of the top 500 most variable probes (**more red the colour the more highly expressed the gene is**) in milk-isolated monocytes (MIMs) at 12, 24, 36, and 48 hpi, respectively, revealed that infected animals separated from control animals in their gene expression response at 36 and 48 hpi except for animal TI2. (G–J) InnateDB network analysis of genes that were differentially expressed at 12, 24, 36, and 48 hpi, respectively (**red** = **upregulated: green** **=** **downregulated**). The networks were visualized in Cytoscape analysis of genes that were differentially expressed at 12, 24, 36, and 48 hpi, respectively. The networks were visualized in Cytoscape.

To investigate the host monocyte transcriptional and posttranscriptional responses to a mastitis-causing pathogen *in vivo*, five Holstein Friesian animals were infected via the teat canal with approximately 500 CFU of *Streptococcus uberis* 0140 in 10 ml saline. Five control animals were inoculated with saline only. Blood and milk samples were taken at 0, 12, 24, 36, and 48 hr postinfection (hpi) and CD14^+^ monocytes were isolated by FACS (Figure 1). The infection was monitored using recorded milk bacterial counts (CFU/ml) and somatic cell counts (per ml) at each of the five time points for each animal (control and infected). On average, bacterial counts peaked in the infected animals at 24 hpi, whereas no change was observed in the uninfected controls. There was, however, significant heterogeneity among the CFU data for each infected animal in terms of the magnitude and the timing of the response (Figure 2A and Table S3). One infected animal (TI3, which had the highest CFU/ml data) peaked at 24 hpi, two others (TI1 and TI4) peaked at 36 hpi, and CFU data for one animal were still climbing at 48 hpi (TI5). Additionally, one infected animal was observed to have only a very modest increase in bacterial counts (TI2). Somatic cell count (SCC) data also confirmed the presence of the infection in the challenged animals and not in the controls. SCC was observed to increase at 24 hpi and by 36 hpi was, on average, >900,000 cells/ml in infected animals. In comparison, the average SCC in control animals at 36 hpi was <52,000 cells/ml (Figure 2B and Table S3). An SCC reading >200,000 cells/ml is generally considered diagnostic of mastitis (Dufour and Dohoo 2013). Again, there was heterogeneity in the SCC response in the infected animals. Interestingly, the infected animal that was observed to have only a modest increase in CFU/ml (TI2) had a relatively robust SCC response.

### Profiling mRNA expression in blood-isolated and milk-isolated CD14^+^ monocytes

NGS approach was applied to monitor the transcriptional (mRNAseq) and posttranscriptional (miRNAseq) changes that occurred in blood-isolated and milk-isolated CD14^+^ monocytes in the infected and control animals (Figure 1). Sequencing of 100 mRNA Illumina libraries (*i.e.*, 50 blood and 50 milk monocyte mRNA libraries) yielded more than four billion sequence reads. More than three billion reads of these mapped uniquely to the *Bos taurus* UMD 3.1 genome (Table S2). The average correlation coefficient of mRNA normalized read counts between samples at each time point was 0.95 for control samples and 0.92 for infected samples, indicating very high reproducibility of the data among replicates (Table S4).

Hierarchical clustering of normalized mRNA read counts from MIMs revealed that the control and infected animals clearly separated at 36 and 48 hpi, except for the one infected sample (TI2) that had very low bacterial counts and likely did not develop a full infection (Figure 2, C–F). This sample was subsequently excluded from differential gene expression analysis. Hierarchical clustering of the normalized mRNA read counts of genes that were DE in blood-isolated monocytes (BIMs) revealed that only three of the infected animals (TI1, TI3, and TI4) separated from uninfected controls at 36 and 48 hpi (Figure S1). These animals also had the highest SCC data at these time points.

We utilized the EdgeR statistical package (Robinson *et al.* 2010) to determine which mRNAs were significantly DE in MIMs and BIMs in response to *S. uberis*. In MIMs, there were 4, 36, 1774, and 1532 upregulated genes at 12, 24, 36, and 48 hpi, respectively. The majority (1254) of genes upregulated at 48 hpi were also upregulated at 36 hpi. Additionally, there were 5, 2, 1518, and 995 downregulated genes in MIMs at those time points. Of the 995 downregulated genes at 48 hpi, 80% were also downregulated at 36 hpi. Overall, 2056 different genes were upregulated and 1721 different genes were downregulated for at least one time point in MIMs in response to *S. uberis* infection (Table S5).

Traditionally, mastitis has been thought of as a local bacterial infection with a robust inflammatory response. In BIMs, however, we observed a much more subtle but still quite significant response to *S. uberis* infection. Only 10 genes were upregulated in BIMs at 36 hpi, but this increased to 83 genes by 48 hpi (Table S5). Nine of the 10 36 hpi genes were also upregulated at 48 hpi. Additionally, 3, 4, 26, and 39 genes were downregulated in BIMs at 12, 24, 36, and 48 hpi, respectively.

### Pathway analysis reveals the suppression of metabolic pathways and the upregulation of inflammatory pathways in response to *S. uberis* infection

**Figure 3:**
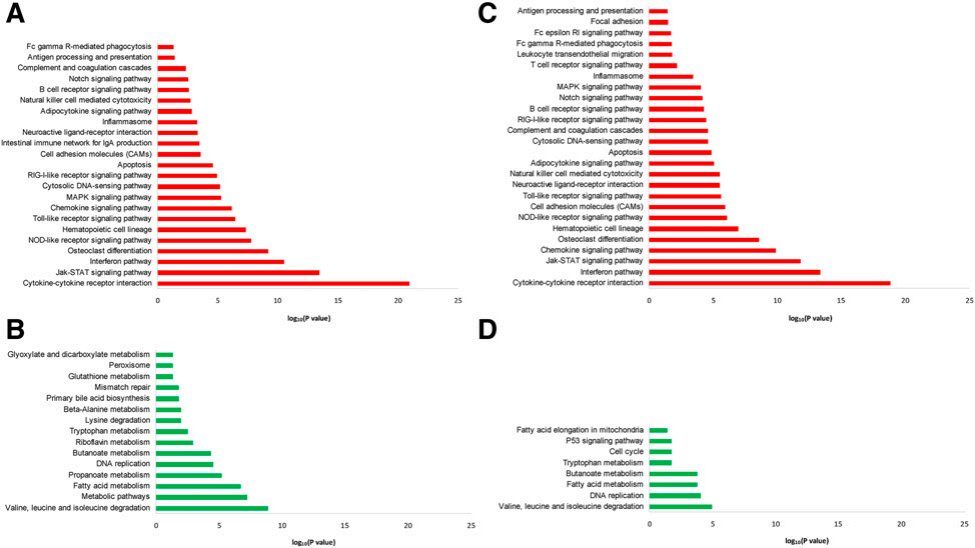
Pathways that were statistically overrepresented among genes. (A) Upregulated at 36 hpi, (B) upregulated at 48 hpi, (C) downregulated at 36 hpi, and (D) downregulated at 48 hpi in MIMs isolated from *S. uberis*–infected animals. Upregulated genes were significantly enriched for roles in inflammatory and other innate immune pathways, whereas downregulated genes were significantly associated with metabolic pathways.

**Figure 4:**
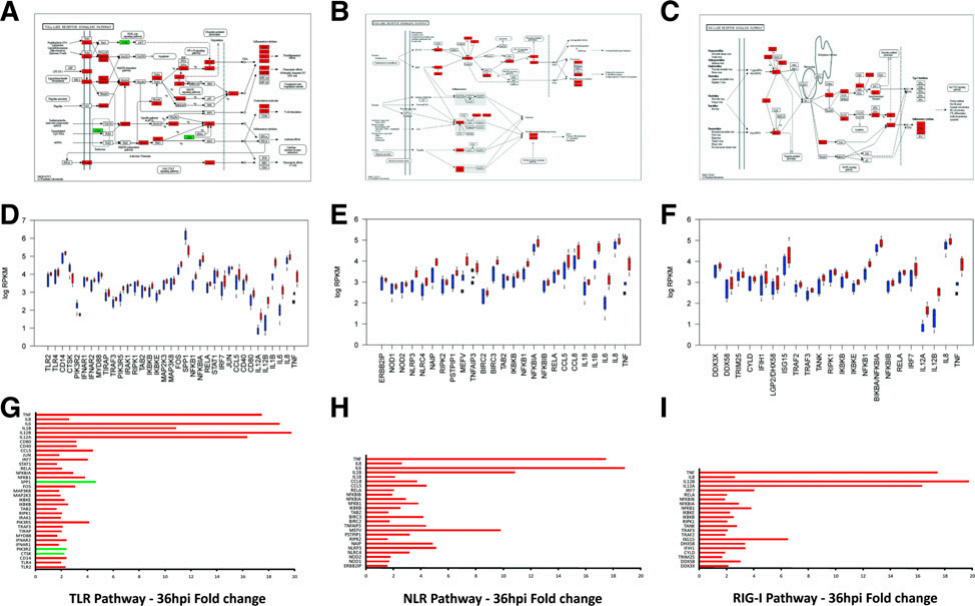
Differential gene expression in key innate immune pathways in MIMs from *S. uberis*–infected animals. (A–C) Differentially expressed (DE) genes highlighted on KEGG Toll-like receptor (TLR), NOD-like receptor (NLR), and RIG-I signaling pathway diagrams (red = upregulated; green = downregulated). (D–F) RPKM values for each of the DE genes in the TLR, NLR, and RIG-I pathways, respectively (red = infected; blue = control). (C) Fold change values for each of the DE genes in the TLR, NLR, and RIG-I pathways, respectively (red = upregulated; green = downregulated).

Pathway analysis of upregulated and downregulated genes at each time point was undertaken using GOseq (Young *et al.* 2010) with pathway annotation imported from the KEGG database (Kanehisa *et al.* 2007) to identify which pathways were statistically overrepresented among DE genes in MIMs and BIMs. Two manually curated pathways (interferon signaling pathway and the inflammasome pathway) were also included (see *Materials and Methods*). No significant pathways were identified among either the BIM or the MIM DE genes at 12 or 24 hpi. At 36 and 48 hpi, however, more than 20 different pathways were identified as being statistically overrepresented (Figure 3 and Table S6). In MIMs, downregulated genes were predominantly associated with metabolic pathways (Figure S2), such as fatty acid and amino acid metabolism, the citric acid (TCA) cycle and glutathione metabolism, as well as DNA replication and repair pathways and the cell cycle. Downregulated pathways were largely similar between 36 hpi and 48 hpi, although fewer pathways were significant at 48 hpi. Upregulated genes, however, were primarily associated with well-known pattern recognition receptor pathways (Figure 4), including the Toll-like receptor pathway [*e.g.*, TLR2, TLR4, CD14, MYD88, TIRAP, and IL-1 receptor-associated kinase 1 (IRAK1) all upregulated], the NOD-like receptor pathway [*e.g.*, NOD1, NOD2, NLRP3 (NALP3), NLRC4 (IPAF), NAIP (NAIP5) upregulated], and the RIG-I-like receptor pathway [*e.g.*, DDX58 (RIG-I), IFIH1 (MDA5), CYLD, DHX58 (LGP2), DDX3X, TRIM25], and interferon signaling and cytokine and chemokine signaling pathways. Upregulated inflammatory cytokine and chemokine genes included the genes encoding TNF, IL-1A, IL-1B, IL-6, IL-8, IL-12A and IL-12B, IL-17B and IL-17C, IL-18, IL-23A, IL-27, CCL3 (MIP1α), CCL4 (MIP1β), CCL5 (RANTES), CCL8 (MCP-2), and CCL20 (MIP3A). The genes encoding TNF, IL-1B, IL-6, IL-12, and CCL20 were more than 10-fold upregulated at 36 hpi. All upregulated pathways that were significant at 36 hpi were still significant at 48 hpi, with five additional upregulated pathways being significant only at 48 hpi. These pathways were primarily related to leukocyte migration and phagocytosis.

In BIMs, only two pathways were statistically overrepresented among 48 hpi upregulated genes, interferon signaling and cytokine–cytokine receptor interaction (Table S6). No pathways were significant at the other time points. Among downregulated genes, there were also few overrepresented pathways. Those pathways that were significant were primarily related to the complement and focal adhesion pathways.

### Network analysis of differentially expressed genes

InnateDB was used to generate a network of experimentally supported molecular interactions that have been annotated to occur directly between the DE genes and their encoded products. Gene expression data from each of the four postinfection time points were then overlaid on this network and the network was visualized using Cytoscape 2.8.2 (Shannon *et al.* 2003) (Figure 2, G J). The network consisted of 2185 nodes (representing DE genes and their encoded products) and 10,786 edges (representing annotated molecular interactions) between them.

**Figure 5:**
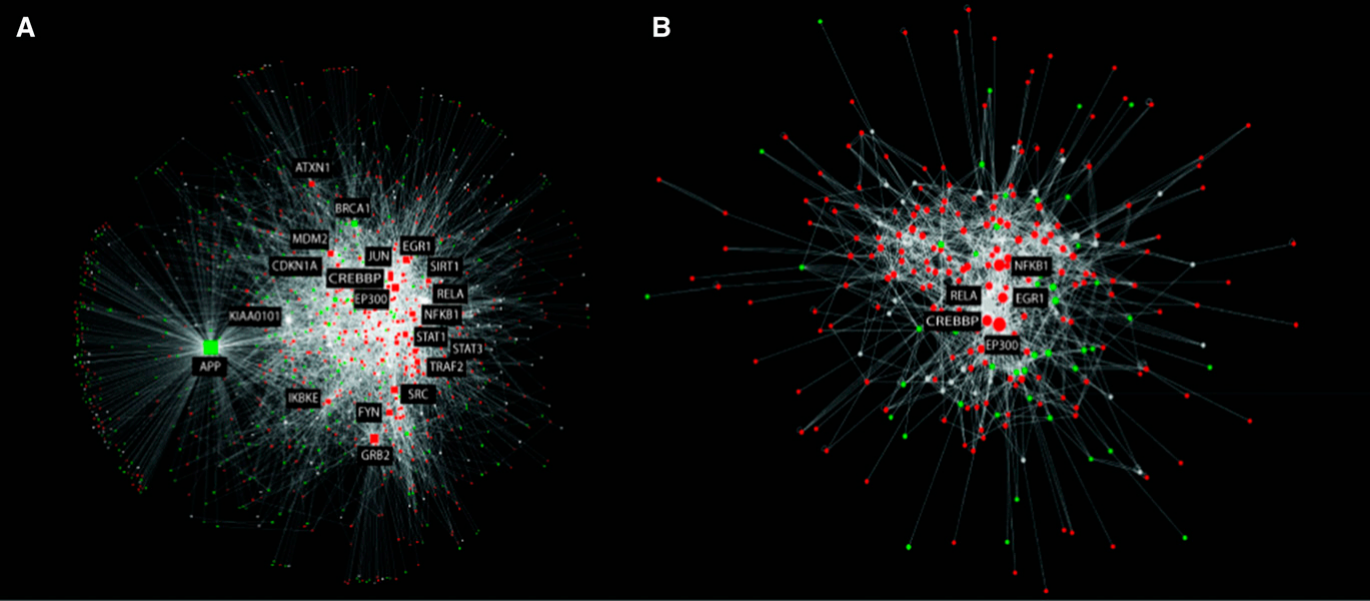
Network analysis of differentially expressed (DE) genes. InnateDB was used to generate a network of experimentally supported molecular interactions that have been annotated to occur directly between the DE genes and their encoded products. (A) The top 20 CytoHubba-predicted network hubs and their interactors. Overlain on the network is gene expression data from MIMs at 36 hpi (red = upregulated; green = downregulated). The top 20 hubs were highly enriched for roles in innate immunity. (B) The high-scoring DE subnetwork identified in the larger network using jActiveModules. This module was highly enriched for several innate immune relevant pathways.

The network was then analyzed using cytoHubba (Lin *et al.* 2008) to identify network hubs and bottlenecks that may represent the key regulatory nodes in the networks. Using the “Degree” algorithm, the top 20 hubs (*i.e.*, genes/proteins that are highly connected to other DE genes) in each network were identified (Table S7). InnateDB Gene Ontology analysis revealed that the top 20 hubs were highly enriched for roles in innate immunity (FDR <1.7e^−8^) and transcriptional regulation (FDR <1.5e^−7^). Many of the top 20 hubs were well-known transcriptional regulators of innate immunity, including JUN, NFKB1, RELA, STAT1, STAT3, EP300, and CREBBP. These transcriptional regulators were located in the most densely connected portion of the network and share many connections (Figure 5A). One interpretation of this is that transcriptional regulation by several of these transcription factors is required for differential gene expression in response to the infection.

Bottlenecks are network nodes that are the key connector proteins in a network and have many “shortest paths” going through them, similar to bridges or tunnels on a highway map (Yu *et al.* 2007). Seventeen of the top 20 hubs were also identified as network bottlenecks. Beta-actin, the transcriptional regulator, FOS (AP1), and ISG15 were additionally identified in the top 20 bottlenecks. ISG15 has been shown to act as a negative regulator of both the NF-κB and RIG-I signaling pathways (Arnaud *et al.* 2011; Minakawa *et al.* 2008).

Identifying hubs solely based on their degree can identify nodes that, in general, have been annotated to have many interactions. This fails to address whether the number of connections in a particular network of interest is more or less than is expected (given the number of known interactions for that node in the database and the size of the network). To address this issue, we have developed the Contextual Hub Analysis Tool (CHAT) (H., L. Wiencko, K., Bryan, A., Noyelle-Depierre & D., J. Lynn., unpublished results ), a network analysis tool that identifies nodes in a network that are more highly connected to contextually relevant nodes (in this case DE nodes) than is expected by chance (Table S8). Applying this method to our network identified that several of the hub nodes (*e.g.*, KIAA0101) did not have more connections to DE genes than expected by chance. This means that these genes, although highly connected, are less likely to be functionally important in our network. However, all of the transcriptional regulators of innate immunity that were identified as hubs in the analysis above (JUN, NFKB1, RELA, STAT1, STAT3, EP300, CREBBP), were also identified by CHAT to be significantly more connected to 36 hpi DE genes than expected by chance. CHAT also identified several other known innate immunity transcriptional regulators in the top 20 "contextual’ hubs, including REL, IRF1, and IRF9. Fifteen of the top 20 contextual hubs are annotated by InnateDB as having a role in innate immunity (FDR <1.16E^−12^). Although the ranking of the top contextual hubs changed from 36 to 48 hpi, the most significant hubs remained the same.

The network was also analyzed using the jActiveModules plugin (Ideker *et al.* 2002) in Cytoscape 2.8.2 (Shannon *et al.* 2003) to identify high-scoring DE subnetworks. This type of analysis can aid in the identification of functionally relevant groups of DE genes that may be acting in concert. A single highly connected component (>10 nodes) was identified consisting of 278 nodes and 1585 interactions. This module consisted of several of the transcriptional hubs (CREBBP, EGR1, EP300, NFKB1, RELA) identified in the analysis above and their interactors (Figure 5B). Pathway analysis of genes in the module revealed that the module was statistically enriched for many of the same pathways that were identified in the analysis of all upregulated genes including Jak-STAT signaling, the TLR, NLR, and RIG-I pathways, apoptosis, and chemokine and cytokine signaling (Table S9).

### Profiling miRNA expression in blood-isolated and milk-isolated CD14^+^ monocytes

Sequencing of 100 miRNA Illumina libraries yielded more than one billion reads for both the MIM and BIM samples. Following a pipeline of quality filtering and adaptor trimming, a total of 312 and 492 million reads from MIMs and BIMs, respectively, mapped uniquely to the *Bos taurus* UMD 3.1 genome (Table S2). Uniquely aligning reads were then assigned to known mRNAs/miRNAs using HTseq based on miRBase v19 annotation of the bovine genome to generate read counts per mature miRNA in each sample (Flicek *et al.* 2012; Hubbard *et al.* 2009). On average, 79% of MIM reads and 80% of BIM reads uniquely mapped to known miRNAs (Figure S3).

RNAseq profiling revealed that the miRNAome of MIMs and BIMs were broadly similar and exhibited a range of miRNA expression that has been observed in many other cell types; 297 and 282 miRNAs were expressed at a threshold of >1 rpm in MIMs and BIMs, respectively (Table S10). Of these, 136 and 116, respectively, were expressed at a level >100 rpm, a level of expression that has been shown to be associated with functional miRNAs (Mullokandov *et al.* 2012).

### Multiple miRNAs are differentially expressed in response to *S. uberis* infection

**Figure 6:**
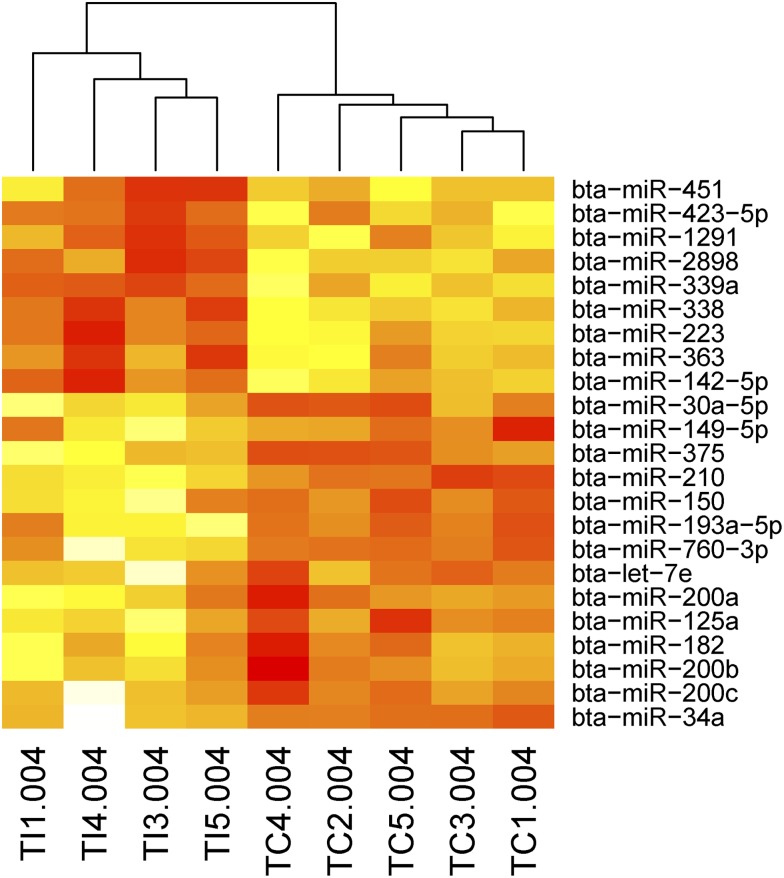
A heatmap of the normalized read counts of miRNAs that were identified as differentially expressed in MIMs at 36 hpi. Hierarchical clustering revealed the separation of the infected and control animals based on this miRNA expression data (**more red the colour the more highly expressed the miRNA is**). Note that one infected animal (TI2) is not included in this analysis because it did not appear to respond to the infection as measured by CFU or mRNA expression data (see Figure 2).

The EdgeR statistical package was utilized to determine which miRNAs were significantly DE in response to *S. uberis* infection at 12, 24, 36, and 48 hpi. To address any normalization issues, we retained only those miRNAs that were robust to the normalization procedure used (Lawless *et al.* 2013). Additionally, only miRNAs that had an average expression of >10 rpm across samples were included for further analysis. In MIMs, we identified that 26 unique miRNAs were DE. Twelve of these miRNAs were DE across more than one time point (Table 1). Hierarchical clustering of the normalized read counts for MIM DE miRNAs revealed that the control and infected animals clearly separated at 36 hpi (Figure 6). Very few miRNAs were DE in blood monocytes. We found three in total, one at 24 hpi and three at 48 hpi (one was DE at both time points) (Table 1).

**Table 1:** Fold changes and false discovery rates of DE miRNAs at 12, 24, 36, and 48 hr postinfection in milk-isolated and blood-isolated monocytes

Tissue	Hours After Infection (hpi)	miR Name	Fold Change	False Discovery Rate
MIMs	12	bta-miR-615	16.53	0.002591533
MIMs	12	bta-miR-451	32.73	0.022589776
MIMs	12	bta-miR-486	1.77	0.051194817
MIMs	36	bta-miR-34a	−8.07	0.003853138
MIMs	36	bta-miR-200c	−7.12	0.021524752
MIMs	36	bta-miR-200b	−6.59	0.022403054
MIMs	36	bta-miR-182	−6.43	0.034621112
MIMs	36	bta-miR-125a	−5.86	0.000854468
MIMs	36	bta-miR-200a	−4.90	0.061309129
MIMs	36	bta-let-7e	−4.51	0.046884171
MIMs	36	bta-miR-760-3p	−4.38	0.030789676
MIMs	36	bta-miR-193a-5p	−4.13	0.099288316
MIMs	36	bta-miR-150	−4.05	0.014886528
MIMs	36	bta-miR-210	−4.03	0.000399613
MIMs	36	bta-miR-375	−3.88	0.021524752
MIMs	36	bta-miR-149-5p	−2.77	0.099288316
MIMs	36	bta-miR-30a-5p	−2.48	0.046884171
MIMs	36	bta-miR-142-5p	2.21	0.033031656
MIMs	36	bta-miR-363	2.23	0.049537718
MIMs	36	bta-miR-223	2.44	0.011440656
MIMs	36	bta-miR-338	2.51	0.017723606
MIMs	36	bta-miR-339a	2.82	0.01613752
MIMs	36	bta-miR-2898	2.98	0.003853138
MIMs	36	bta-miR-1291	3.07	0.033031656
MIMs	36	bta-miR-423-5p	5.73	0.014512876
MIMs	36	bta-miR-451	47.12	6.28E−07
MIMs	48	bta-let-7e	−6.92	0.055709133
MIMs	48	bta-miR-200b	−6.06	0.055709133
MIMs	48	bta-miR-34a	−5.37	0.014991858
MIMs	48	bta-miR-149-5p	−4.01	0.055709133
MIMs	48	bta-miR-375	−3.76	0.006976206
MIMs	48	bta-miR-210	−3.39	0.072510577
MIMs	48	bta-miR-125a	−3.29	0.087523866
MIMs	48	bta-miR-150	−3.18	0.033651802
MIMs	48	bta-miR-99b	−2.9	0.055709133
MIMs	48	bta-miR-338	2.81	0.055709133
MIMs	48	bta-miR-486	12.79	0.006655283
MIMs	48	bta-miR-451	48	1.79E−05
BIMs	24	bta-miR-146b	−23.26	0.023903261
BIMs	48	bta-miR-451	9.74	0.067032312
BIMs	48	bta-miR-146b	−3.52	0.067032312
BIMs	48	bta-miR-411a	−22.44	0.067032312

Many of the miRNAs identified as being DE have been shown to have a role in immunity in other species. miR-223, for example, which was upregulated at 36 hpi in MIMs, has a multifactorial role in neutrophils, regulating their proliferation, activation, and granulopoiesis (Chen *et al.* 2004; Fazi *et al.* 2005). Interestingly, in RAW264.7 cells challenged with lipopolysaccharide (LPS), miR-223 has been reported to be downregulated, allowing the upregulation of signal transducer and activator of transcription 3 (STAT3), which promotes proinflammatory IL-6 and IL-1β transcription. We have previously commented on the differences in the miRNA response to LPS and *S. uberis*, a Gram-positive bacterium (Lawless *et al.* 2013).

Other DE miRNAs in our study that have a demonstrated role in immunity and infection in other species include let-7e, which was downregulated in MIMs after *S. uberis* infection. Let-7e has been shown to regulate caspase 3 and caspase 7 in human monocyte-derived macrophages infected with *Mycobacterium avium hominissuis* (Sharbati *et al.* 2011). Let-7e, as well as several other DE miRNAs in our study (bta-miR-200c, bta-miR-210, and bta-miR-193a), have also previously been identified as DE in bovine mammary epithelial cells stimulated with *S. uberis in vitro* (Lawless *et al.* 2013).

Another DE miRNA with a role in immune regulation is miR-150, which we observed to be downregulated at both 36 and 48 hpi in MIMs. miR-150 targets MyD88 (upregulated at both 36 and 48 hpi in MIMs), a key regulator of TLR signaling (Ghorpade *et al.* 2013). miR-150 has also been shown to target CXCR4 (Rolland-Turner *et al.* 2013; Tano *et al.* 2011), which was upregulated two-fold at 36 and 48 hpi in MIMs. Finally, one of the miRNAs that was identified as downregulated in BIMs, miR-146b, has been shown to target TNF receptor-associated factor 6 and IRAK1 genes in THP-1 cells stimulated with LPS (Taganov *et al.* 2006).

### Predicted targets of downregulated but not upregulated miRNAs are highly enriched for roles in innate immunity

**Figure 7:**
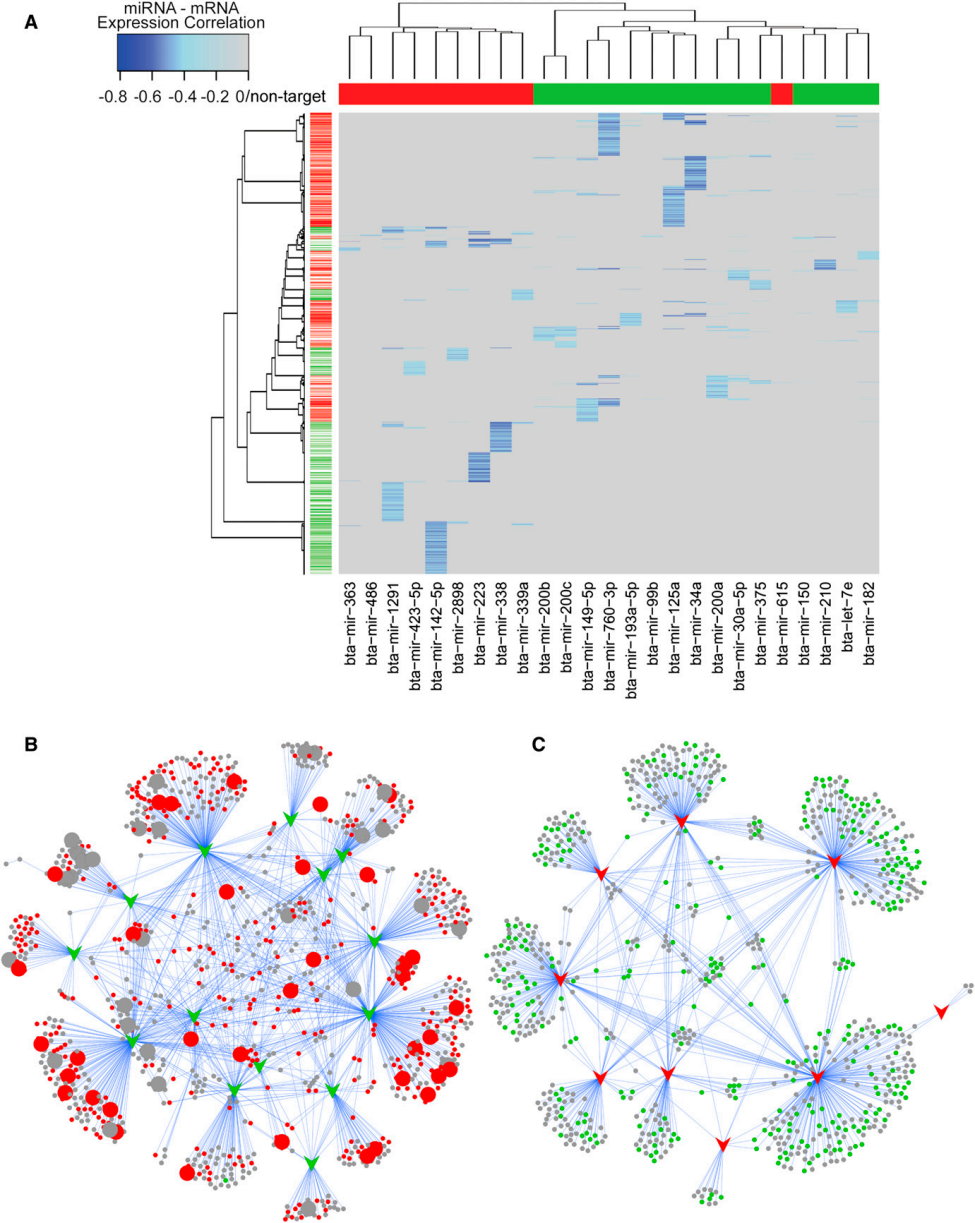
Visualization of miRNA targets. (A) Two-dimensional cluster analysis and heatmap visualization of the miRNA/mRNA expression correlation data matrix after filtering by predicted miRNA-targets and correlation significance. The primary split in the upper hierarchical dendrogram largely aligns with the upregulated miRNA (red) and downregulated miRNA (green). miRNA/mRNA target expression correlations are colored based on increasing significance (light blue to blue) with nontarget/nonsignificant correlations masked (gray). (B) A network representation of the predicted targets (circular nodes) of downregulated miRNAs (green arrows). Red circles = upregulated in MIM mRNA expression data at 36 and/or 48 hpi. Larger circular nodes represent those genes that have been annotated by InnateDB to have a role in innate immunity. (C) A network representation of the predicted targets (circular nodes) of upregulated miRNAs (red arrows). Green circles = downregulated in MIM mRNA expression data at 36 and/or 48 hpi. Note that the predicted targets of downregulated but not upregulated miRNAs are highly enriched for roles in innate immunity.

To identify the potential mRNA targets of DE miRNAs in MIMs isolated from infected animals, we identified those mRNAs whose expression was significantly negatively correlated with miRNA expression. These predictions were further refined by removing those miRNA target–predicted relationships that were not supported by a predicted seed region in the 3′ UTR of the correlated mRNA (Figure 7 and Table S11). Analysis of the predicted target genes using InnateDB (www.innatedb.com) (Lynn *et al.* 2008) revealed that the predicted targets of downregulated but not upregulated miRNAs were highly enriched for roles in innate immunity (FDR <3.2E^−8^). More specifically, pathway analysis revealed that downregulated miRNAs were predicted to preferentially target key pathogen recognition receptor signaling pathways, including the TLR, NLR, and RIG-I signaling pathways (Table S12). Given that these pathways were also identified in the mRNAseq data as being among the top upregulated pathways, this finding strongly suggests that miRNAs are key regulators of innate immune pathways that drive the host inflammatory response during mastitis.

In contrast, the predicted targets of upregulated miRNAs were enriched for roles in metabolism (FDR = 0.01). Pathway analysis of the mRNAseq data had already highlighted the downregulation of metabolic pathways in response to *S. uberis* infection (see above). These results suggest that miRNAs may also be key regulators of the transcriptional suppression of metabolic pathways during mastitis.

### Novel miRNA discovery

The miRNA sequencing data were also mined to determine if milk or blood monocytes expressed potentially novel miRNAs. Previously, we identified 19 novel miRNAs in bovine mammary epithelial cells using miRDeep2 (Lawless *et al.* 2013). Applying the same approach as our previous study, we identified a further 20 high-confidence putatively novel bovine miRNAs that were independently predicted in multiple MIMs/BIMs miRNAseq data (Table S13).

Searching the miRBase database (v20) using BLAST identified that eight of the novel miRNAs had close homology to other miR-2284 family members. miRBase currently lists this miRNA family as having 102 members, yet virtually no data exist regarding what function such a large group of constitutively expressed miRNAs may have. Other novel miRNAs discovered in this study included a homolog of hsa-miR-3680-3p, a miRNA identified in human periphery blood (Vaz *et al.* 2010). The discovery of these miRNA further adds to the database of bovine miRNAs.

## Discussion

Infectious disease is a serious threat not only directly to human health but also to animal health, for which it is associated with substantial annual economic losses, public confidence issues, and food security concerns. Currently, there is a significant gap in the understanding of the molecular and genetic mechanisms that underpin susceptibility to infectious disease in humans and in animals. An important reason for this is the fact that disease susceptibility is a multifactorial complex phenotype, which is not the result of single genes acting in isolation but rather is attributable to perturbation at a network or systems level (Barabasi *et al.* 2011). Such networks are regulated at multiple different levels (*e.g.*, genetic, transcriptional, posttranscriptional) and, as such, a multiomic integrative biology approach is needed to understand them.

Here, we report NGS approach coupled with advanced network and pathway biology methods to simultaneously profile the mRNA and miRNA networks that are differentially regulated *in vivo* in blood-isolated and milk-isolated CD14^+^ monocytes during infection with a bovine mastitis pathogen, *S. uberis*. Bovine mastitis is an inflammation-driven disease of the bovine mammary gland, which costs the global dairy industry billions of dollars per year (Jones and Bailey 2009; Wells *et al.* 1998). Profiling genome-wide changes in mRNA expression in MIMs and BIMs using RNAseq, we observed more than 3500 genes to be statistically altered in their expression in response to *S. uberis* challenge. Notably, this RNAseq approach identified approximately 1000 more DE genes than had been previously reported in a microarray-based analysis of RNA expression in mammary tissue of cattle infected with the same pathogen (Jensen *et al.* 2013; Moyes *et al.* 2009; Swanson *et al.* 2009). As expected, given that mastitis is a relatively localized inflammatory disease in the mammary gland, the majority of DE genes were identified in MIMs, which are recruited to the site of infection. This influx of immune cells (including monocytes) to the site of infection was observed in recorded SCC data only in the infected animals. However, we also observed a small but significant transcriptional response in BIMs to *S. uberis* infection that was primarily associated with an interferon and cytokine signaling signature. Previous studies have also shown more systemic changes in gene expression in neighboring uninfected mammary glands, the liver, and in the blood (Blum *et al.* 2000; Jensen *et al.* 2013; Jiang *et al.* 2008; Mitterhuemer *et al.* 2010).

The predominant signature associated with upregulated mRNAs in MIMs from *S. uberis*–infected animals was the strong transcriptional activation of innate immune and inflammatory gene expression. In particular, we noted the transcriptional activation of key pattern recognition pathways, including the TLR, NLR, and RIG-I pathways, which likely drive the observed proinflammatory response. The involvement of TLR signaling (particularly TLR2 and TLR4) in the host response to mastitis is well-documented (Buitenhuis *et al.* 2011; Ma *et al.* 2011; Mitterhuemer *et al.* 2010; Porcherie *et al.* 2012; Whelehan *et al.* 2011); however, less is known about the involvement of the NLR and RIG-I pathways (Moyes *et al.* 2009). Interestingly, the RIG-I pathway is classically associated with viral RNA recognition; however, recent findings suggest that RIG-I can also recognize nucleic acids released by invasive bacteria and trigger IFN-β and inflammasome activation (Abdullah *et al.* 2012). These findings concur well with the observed interferon and inflammasome activation transcriptional signatures observed in our study.

Several previous *in vitro* studies have strongly suggested roles for miRNAs in regulating bovine immunity (Dilda *et al.* 2011; Lawless *et al.* 2013); however, none of these have globally profiled the miRNA response to infection *in vivo*. In this study, we have also used NGS sequencing to profile miRNA expression in MIMs and BIMs after *S. uberis* infection. Twenty-six miRNAs were identified as DE in MIMs and three were identified in BIMs. Several of these have been previously described as targeting immune or inflammatory regulators in other species. Of particular interest is our finding that downregulated but not upregulated miRNAs in MIMs are predicted to preferentially target genes involved in innate immunity and inflammation. Furthermore, the TLR, NLR, and RIG-I pathways discussed above were all preferentially predicted to be targeted. This strongly suggests that the transcriptional suppression of these miRNAs enables the activation and amplification of the proinflammatory response. Further supporting this conclusion is the fact that several of the DE miRNAs in our study have been validated to target genes in these pathways in other species. miR-149, for example, which was downregulated in MIMs after *S. uberis* infection, has been shown to target mouse CD14 and IRAK1 (Chi *et al.* 2009), key signaling proteins in the TLR pathway. Both CD14 and IRAK1 are also predicted to be targets of bta-miR-149 in our study.

The other predominant transcriptional signature that we found in MIMs after *S. uberis* infection was the widespread repression of a number of metabolic processes (>150 KEGG-annotated metabolism genes are downregulated at 36 hpi). Interestingly, we found that upregulated miRNAs were predicted to preferentially target genes involved in metabolism, suggesting that miRNAs, which are upregulated in response to *S. uberis* infection, may contribute to the transcriptional suppression of metabolic pathways. This signature of metabolic gene transcriptional suppression may appear initially paradoxical in light of the fact that production of inflammatory cytokines is expected to require substantial energy consumption. It is now becoming widely appreciated that activated macrophages undergo the Warburg effect, switching their metabolism from oxidative phosphorylation to glycolysis (McGettrick and O’Neill 2013). This metabolic switch has recently been investigated using a combined metabolomics and microarray approach (Tannahill *et al.* 2013) and has revealed the upregulation of a number of genes involved in glycolysis in bone marrow–derived macrophages challenged with LPS [*e.g.*, solute carrier family 2 (facilitated glucose transporter), member 1 (SLC2A1/GLUT1), hexokinase 3 (HK3), fructose-2,6-biphosphatase 3 (PFKFB3)] and the downregulation of several key genes encoding enzymes in the TCA cycle [*e.g.*, malate dehydrogenase 1 (MDH1) and isocitrate dehydrogenase 2 (IDH2)]. Our transcriptional data are consistent with the data presented in this article (*e.g.*, SLC2A1; HK2 and HK3 and PFKFB4 are all upregulated and MDH1 and IDH1 are downregulated in MIMs at 36 hpi) and suggest that although there is a broad signature of transcriptional suppression of metabolism, these cells are likely to be highly glycolytically active. Several other genes encoding enzymes in the TCA cycle (which was statistically overrepresented among downregulated genes) were also transcriptionally repressed in MIMs at 36 hpi, including the following: dihydrolipoamide dehydrogenase; fumarate hydratase; IDH3B; pyruvate dehydrogenase beta; MDH2; succinate dehydrogenase complex, subunit B; and succinate-CoA ligase, alpha subunit. As has also recently been shown in bone marrow–derived macrophages, LPS strongly increases levels of succinate, a TCA cycle intermediate (Tannahill *et al.* 2013). Succinate acts as an inflammatory signal in macrophages inducing IL1B through the transcription factor HIF-1α, both of which are transcriptionally activated in MIMs 36 hpi (*IL1B* is 10-fold upregulated). Another metabolic pathway that is significantly transcriptionally repressed in MIMs at 36 and 48 hpi is the KEGG Valine, leucine, and isoleucine degradation pathway. Valine, leucine, and isoleucine are branch-chain amino acids that are converted into Acyl-CoA derivatives. These are converted into either acetyl-CoA or succinyl-CoA and enter the TCA cycle (Sears *et al.* 2009). Most of the other significantly downregulated pathways, including fatty acid metabolism, propanoate metabolism, butanoate metabolism, tryptophan metabolism, beta-alanine metabolism, lysine degradation, and glyoxylate and dicarboxylate metabolism, also result in the production of acetyl-CoA and succinyl-CoA that enter the TCA cycle. A similar pattern of expression leading to the transcriptional downregulation of the TCA cycle and alternative pathways involved in producing TCA cycle components has also been reported in other infection models (Chin *et al.* 2010). The transcriptional repression of these pathways is therefore also consistent with a switch in metabolism from oxidative phosphorylation to glycolysis during the proinflammatory response.

Another pathway that is significantly downregulated is the KEGG primary bile acid biosynthesis pathway. Despite the potentially misleading name, this pathway primarily consists of the reactions involved in cholesterol metabolism. The downregulation of genes involved in cholesterol metabolism has also been reported in monocytes isolated from HIV^+^ individuals (Feeney *et al.* 2013) and a number of bacterial infections (Dushkin 2012). The accumulation of cholesterol in monocytes and macrophages leads to the formation of foam cells, which in humans are associated with the inflammatory disease, atherosclerosis (Ross 1999). The downregulation of genes involved in cholesterol and fatty acid metabolism is likely driven in part by the transcriptional suppression of the PPAR-γ transcription factor in MIMs at 36 hpi and the downregulation of PPAR-α at 48 hpi, both of which are key transcriptional regulators of these pathways (Dushkin 2012). Interestingly, the PPARs also have a role in the regulation of inflammation, in which their suppression is required to induce inflammatory gene expression (Bensinger and Tontonoz 2008). Of further note is that one of the liver X receptors, LXR-β (NR1H2), which together with the PPARs is a key regulator of inflammation and lipid metabolism (Bensinger and Tontonoz 2008), is upregulated in MIMs at 36 and 48 hpi. LXRs are also known to antagonize inflammatory gene expression, so it is somewhat surprising to find LXR-β to be upregulated. This may reflect the fact that balance is needed to avoid excessive inflammation or that LXRs and PPARs do not completely overlap in the genes that they regulate.

An additional link between metabolism and inflammation that is currently undergoing intensive investigation is the role of NAD^+^, sirtuins (SIRTs), and AMP-dependent protein kinase (AMPK) in suppressing inflammation (McGettrick and O’Neill 2013). The activation of TLR4, which along with TLR2 and TLR9 is transcriptionally upregulated in MIMs at 36 hpi, has been shown to induce NAM phosphoribosyltransferase (NAMPT; upregulated in MIMs at 36hpi), which in turn activates SIRT1 (upregulated in MIMs at 36 hpi) via NAD^+^. SIRT1 limits inflammation by repressing RELA transcription factor activity, a key transcriptional hub identified in MIMs. SIRT1 also activates AMPK (upregulated in MIMs at 36 hpi), a central regulator of energy metabolism. Activation of AMPK has been shown to decrease NF-κB activity and TNF-α production in macrophages stimulated with LPS, IL-12 production in DCs, and HIF-1α. These data suggest that at 36 hpi, the brakes are starting to be applied to limit the inflammatory response to *S. uberis* infection via SIRT1 and AMPK. The affect of this break is apparent at 48 hpi, where the genes encoding TNF-α, IL-1B, IL-12, and HIF-1α are all downregulated in comparison to 36 hpi. The limiting of inflammation at this stage makes sense based on the bacterial count data, which show that bacterial CFU/ml are declining, suggesting that the infection is being resolved. Interestingly, AMPK activity also inhibits both the cholesterol and fatty acid metabolic pathways that, as discussed above, are downregulated in MIMs after *S. uberis* infection. This suppression of fatty acid metabolism has been shown to be beneficial to the host during a viral infection (Moser *et al.* 2012).

Finally, we also observed the statistically significant over-representation of downregulated genes annotated in the KEGG glutathione metabolism pathway. Glutathione has an important role in innate and adaptive immunity and has been shown to confer protection against microbial, viral, and parasitic infections (Morris *et al.* 2013). Glutathione metabolism also plays an important role in macrophages in the detoxification of reactive oxygen species. The transcriptional suppression of this pathway in MIMs after *S. uberis* infection may be a consequence of the metabolic switch to glycolysis and may be detrimental to the host, leading to an excess of oxidants in the cells, which could drive the inflammation and tissue damage that are characteristic of mastitis. In patients with active tuberculosis, PBMC intracellular glutathione levels declined by 70%; this was correlated with increased proinflammatory cytokines and enhanced bacterial growth (Guerra *et al.* 2012). Supplementation with glutathione has been demonstrated to lead to the control of mycobacterial growth (Venketaraman *et al.* 2003) and also appears to have beneficial effects in reducing inflammation in HIV^+^ patients (Morris *et al.* 2012). This suggests that glutathione supplementation is a potential strategy to reduce the effects of inflammation in mastitis.

miRNAs also likely play a key role in regulating the links between inflammation and metabolism observed in this study. Human SIRT1, for example, which as discussed above limits inflammation, has been shown to be a target of miR-34a (Yamakuchi *et al.* 2008). Our data are consistent with this relationship also existing in MIMs, where we have found bta-miR-34a to be downregulated at 36 and 48 hpi and SIRT1 to be upregulated. Other examples of miRNAs that likely transcriptionally regulate metabolic pathways in MIMs include miR-451, which is upregulated in MIMs at 36 and 48 hpi and has been shown to target MO25 in mouse heart tissue altering AMPK signaling (Chen *et al.* 2012). miR-451 has also been shown to regulate the expression of several proinflammatory cytokines in mice in response to influenza infection (Rosenberger *et al.* 2012).

Aside from providing new insight into the regulatory role miRNAs play in *S. uberis* infection *in vivo*, our study also provides the groundwork for a number of potential practical applications in veterinary medicine. miRNAs, for example, exhibit many properties that have made them of significant interest as noninvasive biomarkers. miRNAs are abundantly and stably expressed in a range of accessible tissues, including serum, milk, urine, saliva, and semen, where they can be readily measured (Chen *et al.* 2010; Hata *et al.* 2010; Kosaka *et al.* 2010). Importantly, for a potential biomarker, miRNAs have high information content, and the expression profile of small numbers of them have been shown to be diagnostic of disease (De Guire *et al.* 2013). The use of miRNAs as a clinical biomarker is most advanced in human cancer research. In 2009, Prometheus Laboratories released an miRNA biomarker to accurately identify 25 different tumor types (Ajit 2012), and miRNA biomarkers are now available for early cancer prognosis from two other companies, Asuragen and Rosetta Genomics. NGS-based technologies, such as the approach used in this study, are empowering RNA expression profiling, including miRNAs, on a genome-wide scale with unprecedented resolution, accuracy, and speed and at a relatively low cost (Schuster 2008). There is significant potential to develop these approaches as diagnostics of infection in animals and also in humans.

## Supplementary Material

Supporting Information
