# Supplementary material for: MicroRNA Regulation of Bovine Monocyte Inflammatory and Metabolic Networks in an In Vivo Infection Model
Source: G3 (Bethesda). 2014 Jan 23;4(6):957–71. doi: 10.1534/g3.113.009936 (PMC4065264; doi:10.1534/g3.113.009936)
Supplement: Supporting Information [file supp_4_6_957__index.html]

MicroRNA Regulation of Bovine Monocyte Inflammatory and Metabolic Networks in an In Vivo Infection Model — Supporting Information 

# MicroRNA Regulation of Bovine Monocyte Inflammatory and Metabolic Networks in an *In Vivo* Infection Model

## Supporting Information for Lawless *et al.*, 2014

**Files in this Data Supplement:**

- Supporting Information - Figures S1-S3, File S1, and Tables S1-S13 (PDF, 722 KB)
- Figure S1 - Heatmap of differential gene expression (tpm) in blood isolated monocytes across infected and control animals at 12, 24, 36, & 48hpi. (PDF, 423 KB)
- Figure S2 - Down-regulated genes highlighted on the KEGG metabolism network. (PDF, 393 KB)
- Figure S3 - The proportion of reads aligning uniquely to bovine ncRNAs. (PDF, 348 KB)
- File S1 - 1.1 MirVanaTM RNA Isolation Kit protocol, 1.2 MirPremierTM microRNA Isolation Kits protocol, 1.3 TruSeq RNA Sample Preparation Kit v2 (50 cycles), 1.4 TruSeq Small RNA Sample Preparation Kit (50 cycles), 1.5 RNA integrity and quantification, 1.6 References. (PDF, 317 KB)
- Table S1 - Manually generated pathway annotations for inflammasome and interferon pathways sourced from SA biosciences (Qiagen) RT2 Profiler™ PCR Array Human Interferon and Receptors (PAHS-064A), and RT2 Profiler™ PCR Array Human Inflammasome (PAHS-097A) annotations. (.xls, 27 KB)
- Table S2 - Summary read statistics and number of unique alignments for each RNA and miRNA library. (.xls, 81 KB)
- Table S3 - Summary of milk volumes, rectal temperatures, ambient temperatures, humidity, and bacterial CFU counts for each animal. (.xls, 63 KB)
- Table S4 - Average correlation coefficients of mRNA normalized read counts between samples at each time-point for control and infected biological replicates. (.xls, 38 KB)
- Table S5 - Differentially expressed genes in milk and blood isolated monocytes at 12, 24, 36, and 48 hours post infection. (.xls, 869 KB)
- Table S6 - Significantly over-represented KEGG pathways among differentially expressed genes in milk and blood isolated monocytes at 12, 24, 36, & 48 hours post infection. (.xls, 51 KB)
- Table S7 - The top 20 network hubs identified using CytoHubba and enriched Gene Ontology terms among hubs. (.xlsx, 13 KB)
- Table S8 - The top 20 contextual hubs identified using the CHAT software. (.xlsx, 65 KB)
- Table S9 - Significantly over-represented KEGG pathways among genes identified in the jActiveModules module. (.xlsx, 10 KB)
- Table S10 - miRNAs that are expressed greater than or equal to 1 tag per million in bovine blood and milk isolated monocytes. (.xls, 324 KB)
- Table S11 - Summary of co-expression, target analysis, and Pearson correlations of miRNA and mRNA data. (.xls, 7 MB)
- Table S12 - Significantly over-represented KEGG pathways among predicted miRNA targets. (.xls, 43 KB)
- Table S13 - Putative novel bovine miRNAs discovered through miRDeep2 analysis of miRNAseq data from 100 milk and blood isolated monocytes. (.xls, 37 KB)
