# Supplementary material for: MicroRNA Regulation of Bovine Monocyte Inflammatory and Metabolic Networks in an In Vivo Infection Model
Source: G3 (Bethesda). 2014 Jan 23;4(6):957–71. doi: 10.1534/g3.113.009936 (PMC4065264; doi:10.1534/g3.113.009936)
Supplement: Supporting Information [file supp_g3.113.009936_FigureS1.pdf]

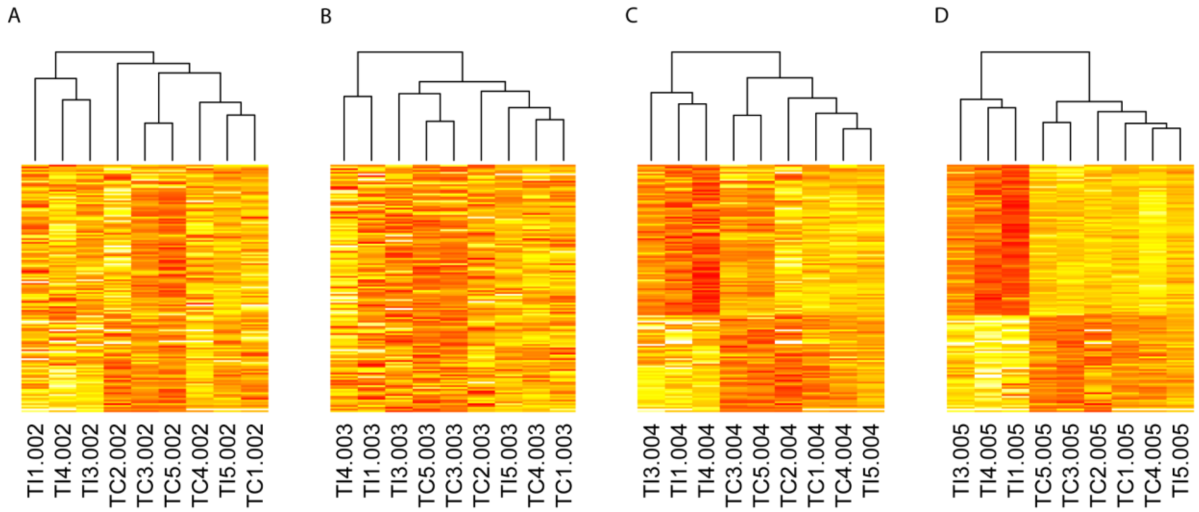

Figure S1 Heatmap of differential gene expression (tpm) in blood isolated monocytes across infected and control animals at 12, 24, 36, & 48hpi. The more red the color the more highly expressed that gene is, R (V2.15.2) hclust package.
