# Supplementary figures and images for: MicroRNA Regulation of Bovine Monocyte Inflammatory and Metabolic Networks in an In Vivo Infection Model
Source: G3 (Bethesda). 2014 Jan 23;4(6):957–71. doi: 10.1534/g3.113.009936 (PMC4065264; doi:10.1534/g3.113.009936)

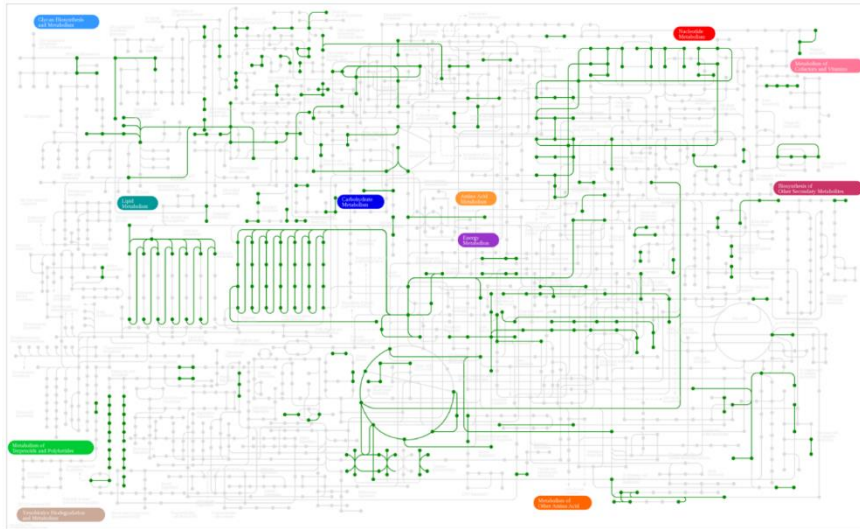

**Figure S2** Down-regulated genes highlighted on the KEGG metabolism network.

Supplement: Supporting Information [file supp_g3.113.009936_FigureS2.pdf]
