# Supplementary material for: MicroRNA Regulation of Bovine Monocyte Inflammatory and Metabolic Networks in an In Vivo Infection Model
Source: G3 (Bethesda). 2014 Jan 23;4(6):957–71. doi: 10.1534/g3.113.009936 (PMC4065264; doi:10.1534/g3.113.009936)
Supplement: Supporting Information [file supp_g3.113.009936_FigureS3.pdf]

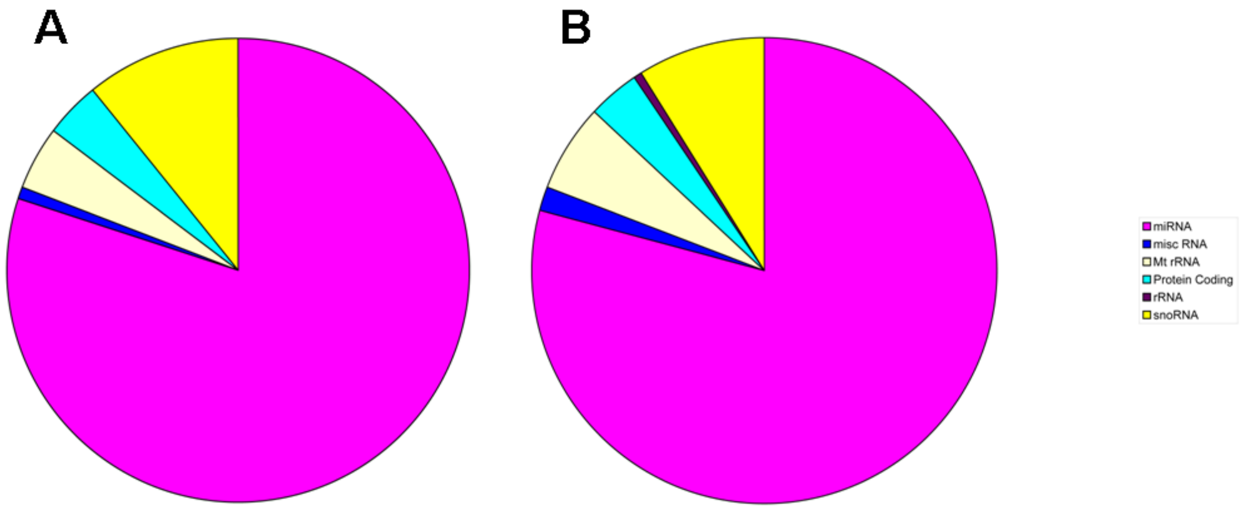

Figure S3 The proportion of reads aligning uniquely to bovine ncRNAs. A) Reads aligning to ncRNAs in milk isolated monocytes. B) Reads aligning to ncRNAs in blood isolated monocytes. The majority of reads align to known miRNAs.
