# Supplementary material for: MicroRNA Regulation of Bovine Monocyte Inflammatory and Metabolic Networks in an In Vivo Infection Model
Source: G3 (Bethesda). 2014 Jan 23;4(6):957–71. doi: 10.1534/g3.113.009936 (PMC4065264; doi:10.1534/g3.113.009936)
Supplement: Supporting Information [file supp_g3.113.009936_FileS1.pdf]

File S1

Table of contents

1.1 MirVanaTM RNA Isolation Kit protocol.

1.2 MirPremierTM microRNA Isolation Kits protocol.

1.3 TruSeq RNA Sample Preparation Kit v2 (50 cycles).

1.4 TruSeq Small RNA Sample Preparation Kit (50 cycles).

1.5 RNA integrity and quantification.

1.6 References.

1.1 MirVana™ RNA Isolation Kit protocol.

Total RNA samples were prepared independently for 50 blood-isolated CD14+ monocyte samples and 50 milk-isolated CD14+ monocyte samples. For each sample, an initial volume of 600 µL of lysis/binding solution (provided with kit) was added to the cells and mixed by vortex to fully disrupt cells and form a homogenous lysate. One volume of acid-phenol: chloroform equal to the lysate volume was then added and the sample was mixed by vortex for 30-60 seconds. The sample was then centrifuged for 5 min at 10,000 x g at room temperature to separate the aqueous and organic phases. The aqueous (upper) phase was removed and transferred to a fresh tube. 1.25 volumes of 100% ethanol were added to the aqueous phase recovered from the organic extraction and mixed thoroughly by vortex. The lysate/ethanol mixture was then pipetted onto the filter cartridge (provided with kit) and centrifuged at 10,000 x g for ~15 sec to pass the mixture through the filter. The flow-through was discarded, and 700 µL wash solution 1 (working solution mixed with ethanol) (Pharmco-AAPER, Brookfield, CT, USA.) added to the filter cartridge and centrifuged for ~5-10 sec. Again, the flow-through from the collection tube was discarded, and the filter replaced in the cartridge as in the previous step. 500 µL wash solution 2/3 (working solution mixed with ethanol) was then added to the filter cartridge and centrifuged for ~5-10 seconds. This step was then repeated. After discarding the flow through from the last wash, the filter was replaced in the cartridge in the same collection tube and the assembly was spun down for 1 min to remove residual fluid from the filter. The filter cartridge was transferred into a fresh collection tube (provided with kit.). 50 µL of pre-heated (95°C) nuclease free water was added to the centre of the filter, and centrifuged for ~20-30 sec at max speed to recover the RNA. The purified RNA was stored at -70 °C until needed.

1.2 MirPremier™ microRNA Isolation Kit protocol.

Small RNA samples were prepared independently for the same 50 blood-isolated CD14+ monocyte samples and 50 milk-isolated CD14+ monocyte samples. MicroRNA lysis buffer was made according to the standard Sigma-Aldrich protocol. Briefly, equal volumes of lysis buffer, and binding solution were made up with 10 mL of 2-mercaptoethanol per 1 ml. The cell pellet was shaken for 1-2 seconds to loosen cells. The Lysis Mix was added to the cell pellet and mixed by vortex immediately but gently and briefly (2-3 seconds) to disrupt the cell pellet. The sample was incubated at room temperature for 5 minutes and mixed 2-3 times in between by gentle shaking. The sample was then centrifuged at maximum speed (16,000 x g) in a standard

microcentrifuge for 5 minutes to remove cellular debris, genomic DNA, and large RNA. The supernatant was transferred to a clean 2-ml Collection Tube. 1.1 volumes of 100% ethanol was then added to the clarified lysate for RNA binding, and mixed immediately and thoroughly by vortex or inversion. 700 mL of the mixture was pipetted into a binding column and centrifuged at maximum speed (16,000 x g) for 30 seconds. The flow through was decanted, and the step repeated. 700 mL of 100% ethanol was then added into the column and centrifuged at maximum speed (16,000 x g) for 30 seconds. The binding column was then transferred into a fresh collection tube, into which 500 mL of the Ethanol-diluted Wash Solution 2 (provided by Sigma-Aldrich) was added into the column. The column was then centrifuged at maximum speed (14,000 –16,000 x g) for 30 seconds. The flow-through was discarded and the column returned to the Collection Tube. Another 500 mL of the Ethanol-diluted Wash Solution 2 was added into the column and centrifuged at maximum speed (16,000 x g) for 30 seconds. The flow-through was discarded the column returned to the Collection Tube. To dry the column, the column was centrifuged at maximum speed (16,000 x g) for 1 minute to dry, and carefully removed from the centrifuge to avoid splashing the residual flow through liquid to the dried column. To elute RNA, the column was added to a new 2 ml collection tube and 30 mL of elution solution (nuclease free water) was added directly onto the centre of the filter inside the column, and let sit for 1 minute. The tube was then centrifuged at maximum speed (16,000 x g) for 1 minute to elute. Elute was collected, and step was repeated. The purified small RNA was stored at -70 °C until needed.

1.3 TruSeq RNA Sample Preparation Kit v2 (50 cycles) protocol.

Briefly, magnetic beads were used to purify out poly-A containing mRNA. Once purified, mRNA was then fragmented and primed with random hexamers into first strand cDNA using reverse transcriptase/ random primers. The RNA template was then removed and double stranded cDNA was generated using DNA polymerase I and RNase H. cDNA fragments ends were blunted, and the 3’ ends were adenylated with a single ‘A’ before ligating the adaptors. Samples were then purified and the products with adaptors were selectively enriched by PCR. The finished libraries were validated on an Agilent bioanalyser using an Agilent DNA-1000 chip (Agilent, Colorado Springs, CO, USA), at which point they were loaded for cluster generation. The samples were sequenced on an Illumina HiSeq 2000. Infected and control samples (n =100) were randomised across four flow cells (i.e. 3 or 4 samples multiplexed per lane), to avoid confounding flow cell/lane effects (Auer and Doerge 2010). The barcode compatibility chart provided with the TruSeq RNA sample preparation kit was adhered to when pooling libraries. Fastq files were produced using the CASAVA 1.8 pipeline.

1.4 TruSeq Small RNA Sample Preparation Kit (50 cycles) protocol.

Briefly, the 3’ and 5’ adaptors were sequentially ligated to each sample. cDNAwas made from each sample successfully ligated with both adaptors via reverse transcriptase and enriched by PCR. The amplified cDNA product was purified through a 6% Novex TBE PAGE gel. Samples were re-eluted in 10 mM Tris-HCL, pH 8.5, and pooled based on Illumina recommended multiplexing protocols. The finished libraries were validated on an Agilent bioanalyser using an Agilent DNA high sensitivity chip (Agilent, Colorado Springs, CO, USA), at which point they were loaded for cluster generation. The samples were sequenced on an Illumina HiSeq 2000. Infected and control samples (n =100) were randomised across three flow cells (i.e. 7 or 8 samples multiplexed per lane), to avoid confounding flow cell/lane effects (Auer and Doerge 2010). Fastq files were produced using the CASAVA 1.8 pipeline.

1.5 RNA integrity and quantification.

Total RNA was measured by the Agilent RNA 6000 Nano Kit using the 2100 Bioanalyzer (Agilent Technologies, Colorado Springs, CO, USA). The integrity of each RNA sample was examined before proceeding with experiment. The Agilent small RNA Kit (Agilent Technologies) was used to quantify miRNA. All miRNA samples were above the required quantity for sequencing.

1.6 References.

Auer, P. L. and R. W. Doerge (2010). "Statistical design and analysis of RNA sequencing data." Genetics 185(2): 405-16.
